# Two complementary approaches for intracellular delivery of exogenous enzymes

**DOI:** 10.1038/srep12444

**Published:** 2015-07-24

**Authors:** Aleksander Rust, Hazirah H. A. Hassan, Svetlana Sedelnikova, Dhevahi Niranjan, Guillaume Hautbergue, Shaymaa A. Abbas, Lynda Partridge, David Rice, Thomas Binz, Bazbek Davletov

**Affiliations:** 1 The University of Sheffield, Western Bank, Sheffield, UK; 2 MRC laboratory of Molecular Biology, Hills Road, Cambridge, UK; 3 Medizinische Hochschule Hannover, Carl-Neuberg-Straße, Hannover, Germany

## Abstract

Intracellular delivery of biologically active proteins remains a formidable challenge in biomedical research. Here we show that biomedically relevant enzymes can be delivered into cells using a new DNA transfection reagent, lipofectamine 3000, allowing assessment of their intracellular functions. We also show that the J774.2 macrophage cell line exhibits unusual intracellular uptake of structurally and functionally distinct enzymes providing a convenient, reagent-free approach for evaluation of intracellular activities of enzymes.

The cell interior is protected by cellular membranes that present an obstacle for the delivery of biological macromolecules into the cytosol for research or therapeutic purposes^1^. Biologicals have become increasingly popular over recent years as small molecule pipelines have decreased^2^. Enzymes have been investigated for therapeutic use due to their highly specific biological activity. The selective and catalytic nature of these enzymes enables them to exert effects on complex cellular processes at low (nanomolar) concentrations not possible with most chemical drugs. The majority of current therapeutic enzymes target extracellular processes contributing to disease; for instance, asparaginases are used in the treatment of leukaemia to decrease serum levels of asparagine^3^ whilst DNases are used to break down extracellular DNA in the treatment of cystic fibrosis^4^. The identification and characterisation of intracellularly active enzymes with therapeutic potential has been hindered by the impermeable nature of cell membranes. Given the great promise enzymes offer for the development of future therapies, new tools that assist investigation of their function in the intracellular environment are highly desirable.

Cells can take up many macromolecules by endocytosis, but these macromolecules are normally channelled into the endolysosomal pathway resulting in their degradation^5^. To overcome this problem a range of techniques to aid the delivery of proteins into the cytosol have been developed. These include physical techniques, such as microinjection and electroporation, and biochemical techniques utilising protein transduction reagents, cell-penetrating and endosome-disrupting peptides^5^,^6^. These techniques, however, often suffer from issues of complexity, low efficiency and cytotoxicity, demanding new approaches^6^. Simple and efficient delivery of proteins into cells remains a bottleneck for assaying intracellular function, greatly limiting the flexibility of experimental design.

We have recently demonstrated that botulinum-derived proteases can be easily delivered into neuroendocrine cells using certain DNA lipofection reagents, revealing a novel action of botulinum enzyme type C in neuroendocrine tumour cells^7^. Here we investigated whether this approach could be applied to other biomedically relevant proteins such as translation-inhibiting enzymes. We used a range of cancer cell lines and show that lipofectamine 3000 efficiently delivers enzymes into the cytosol. Remarkably, a macrophage cell line J774.2 exhibited high sensitivity to extracellular proteins even without lipofection, enabling future high-throughput screening of bioactive enzymes.

## Results

**Figure 1:**
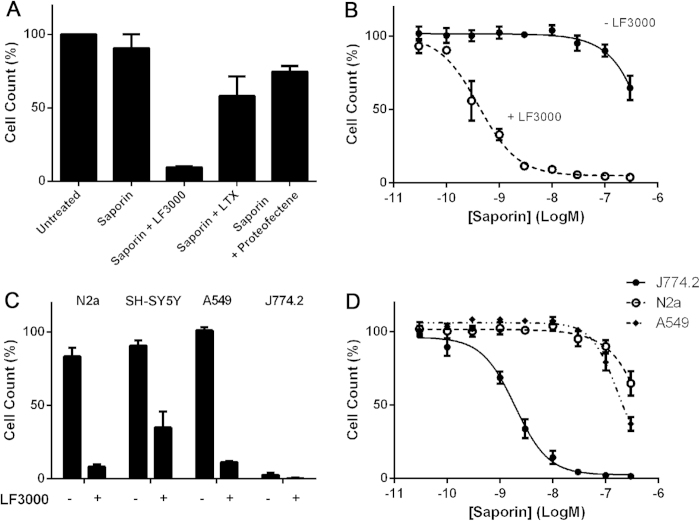
Intracellular delivery of ribosome-inactivating enzyme saporin. (**A**) Saporin (30 nM) potently inhibits growth of neuroblastoma N2a cells in the presence of lipofectamine 3000 (LF3000), but not in the presence of lipofectamine LTX or proteofectene upon 72 hr exposure. (**B**) LF3000 drastically increases sensitivity of the neuroblastoma cells to saporin (EC_50_ = 1 nM with LF3000). (**C**) LF3000 renders the indicated cells sensitive to saporin (30 nM) whereas the macrophage J774.2 cell line exhibits sensitivity even in the absence of LF3000. (**D**) Saporin inhibits growth of the J774.2 cells at low nanomolar concentrations whereas its effect on mouse neuroblastoma N2a cells and human lung cancer A549 cells is evident only above 100 nM concentrations. All experiments were in triplicates, mean ± SEM.

The ribosome-inactivating protein saporin is a plant-derived enzyme which can potently inhibit protein translation and is thus widely used in biomedical research related to cancer and neurological function^8^,^9^. Unmodified saporin cannot enter cells but at high, micromolar concentrations it can trigger apoptosis due to background endocytosis. When saporin is targeted to cell membrane receptors using antibodies or ligands, its cytotoxic effects can be observed at therapeutically relevant, nanomolar concentrations^8^. We tested a range of transfection reagents, including lipofectamine 3000 (LF3000), lipofectamine LTX and proteofectene, a bona-fide protein transfection reagent, for their ability to deliver saporin into mouse neuroblastoma N2a cells. Cell counting using a tetrazolium salt-based assay revealed that saporin at 30 nM concentration, in the absence of any delivery reagent, was not cytotoxic after 72 hrs when compared to the untreated control (Fig. 1A). The presence of the newly-introduced LF3000 led to a dramatic, ten-fold drop in cell survival whilst proteofectene and lipofectamine LTX exhibited only modest effects (Fig. 1A). Titration experiments demonstrated that LF3000 increased the sensitivity of N2a cells to saporin by 1000 fold (Fig. 1B). None of the transfection reagents displayed toxicity on their own.

Next, we investigated whether efficient LF3000-based delivery of saporin can be reproduced in other cancer cell lines: human neuroblastoma SH-SY5Y, human lung adenocarcinoma A549 and mouse macrophage J774.2 cells. The LF3000 significantly enhanced cytoxicity of saporin towards the human neuroblastoma and lung cancer cells. Remarkably, the J774.2 macrophage cell line was sensitive to saporin regardless of the presence of LF3000 (Fig. 1C). Titration experiments with the three cancer cell lines confirmed that J774.2 macrophage cells exhibit an unusual sensitivity to saporin compared to other cells (Fig. 1D).

**Figure 2:**
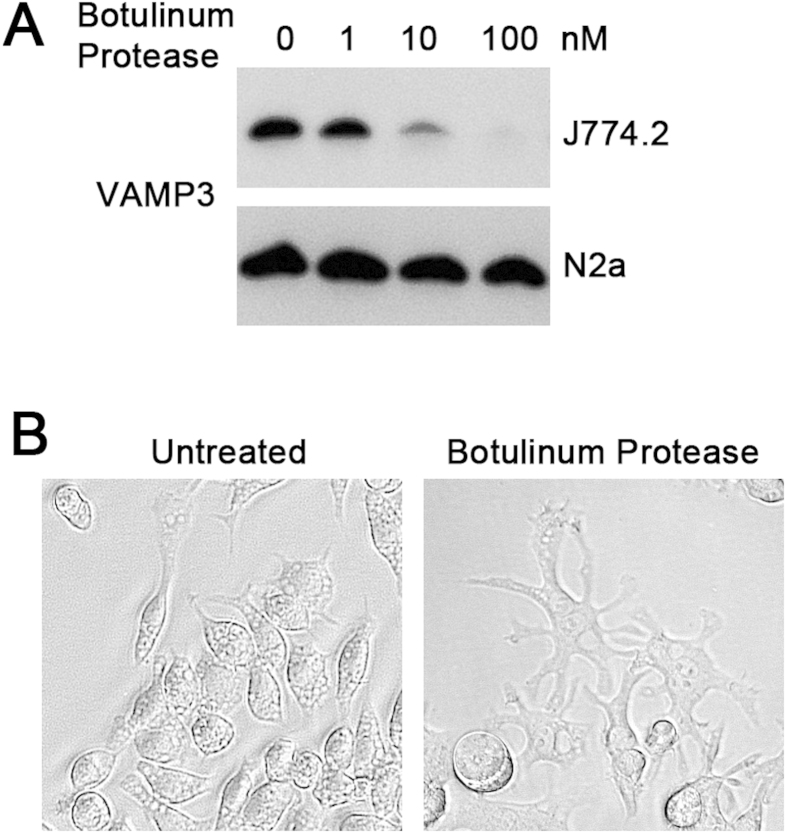
The macrophage J774.2 cell line is sensitive to the botulinum enzyme type D. (**A**) Immunoblot showing cleavage of VAMP3 in J774.2, but not in N2a cells, by botulinum enzyme type D at the indicated concentrations after 72 hrs incubation. (**B**) The J774.2 cells exhibit splayed morphology after 72 hrs incubation in the presence of botulinum enzyme type D (100 nM).

To assess whether J774.2 macrophages are penetrable to structurally/functionally distinct proteins, we analysed biological effects of the enzyme derived from botulinum neurotoxin type D. This protease cleaves the vesicle-associated membrane proteins (VAMPs) involved in membrane trafficking including VAMP3, also known as cellubrevin, which is ubiquitously present in all cells^10^. When the botulinum protease was incubated with J774.2 cells and neuroblastoma N2a cells for 72 hrs, we detected efficient cleavage of VAMP3 by Western immunoblotting only in the J774.2 cells (Fig. 2A). Interestingly, cleavage of VAMP3 resulted in a dramatic change in cell morphology with the macrophage-derived cells adopting a stellate phenotype (Fig. 2B). A similar phenomenon has been observed in previous studies where VAMP3 expression was knocked down using a siRNA approach implicating VAMP3 in cell spreading, adhesion and migration of macrophages^11^. Thus the J774.2 cell line may present a convenient model to study effects of externally applied enzymes on intracellular substrates.

**Figure 3:**
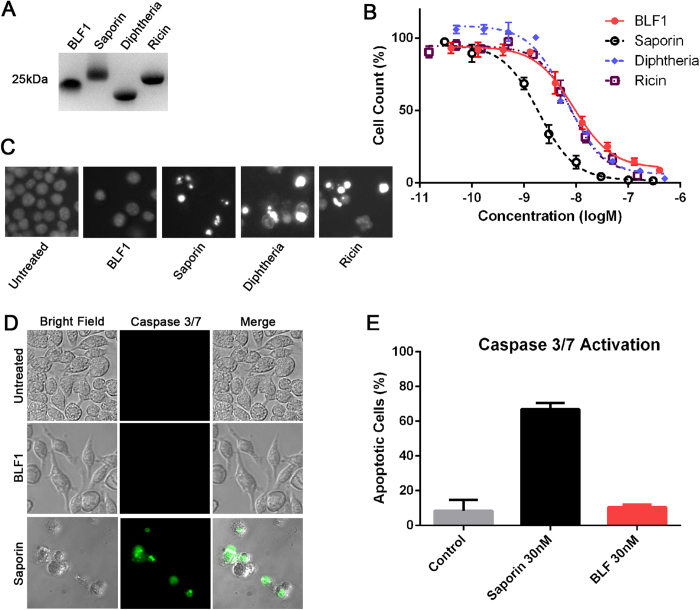
Intracellular effects of translation-inhibiting enzymes on the macrophage J774.2 cell line. (**A**) Coomassie-stained SDS-PAGE gel showing the enzymes used (2 μg per well). (**B**) Translation-inhibiting BLF1, ricin-derived enzyme and diphtheria toxin-derived enzyme exhibit similar efficiency (EC_50_ ≈ 10 nM) whereas saporin blocks cell growth with EC_50_ ≈ 2 nM. (**C**) Hoechst staining reveals that saporin, ricin- and diphtheria-derived enzymes cause chromatin condensation, whereas BLF1 has minimal effects on nuclei; all enzymes tested at 30 nM. (**D**) Representative images of the J774.2 cells stained using caspase 3/7-detecting reagent show apoptosis after treatment with 30 nM saporin but not 30 nM BLF1. (**E**) Bar chart showing that at similar concentrations BLF1 does not cause apoptosis as measured by the fluorescent caspase 3/7 assay. All experiments were in triplicates, mean ± SEM.

Next we used J774.2 cells to compare the efficacy of four enzymes targeting translation mechanisms (Fig. 3A). Among these, ricin-derived enzyme and saporin both cleave the N-glycosidic bond of an adenine in the 28S ribosomal RNA causing a potent block of protein translation^8^. Diphtheria toxin-derived enzyme, on the other hand, transfers ADP-ribose to a histidine of eukaryotic elongation factor 2, inhibiting the polypeptide elongation phase of protein synthesis^12^. Burkholderia lethal factor 1 (BLF1), is a newly discovered enzyme which acts by inhibiting the eukaryotic initiation factor 4A (eIF4A) thereby specifically targeting the initiation step of protein translation^13^. By using the J774.2 cell line, we were able to bypass the need for protein delivery techniques and directly compare the effects of these four enzymes. Titration experiments using the cell proliferation assay showed that BLF1 had a similar inhibitory potency to ricin and diphtheria (EC_50_ ≈ 10 nM) but was five times less efficient than saporin (Fig. 3B). Analysis of nuclear morphology by Hoechst staining revealed that saporin, ricin and diphtheria enzymes all caused chromatin condensation characteristic of apoptosis, whereas BLF1 appeared to inhibit proliferation with minimal effects on nuclei at low nanomolar concentrations (Fig. 3C). Further analysis using a fluorescent caspase 3/7-based assay confirmed that saporin induces apoptosis at low nanomolar levels whereas the J774.2 cells survived treatment by BLF1 at a similar concentration (Fig. 3D,E).

**Figure 4:**
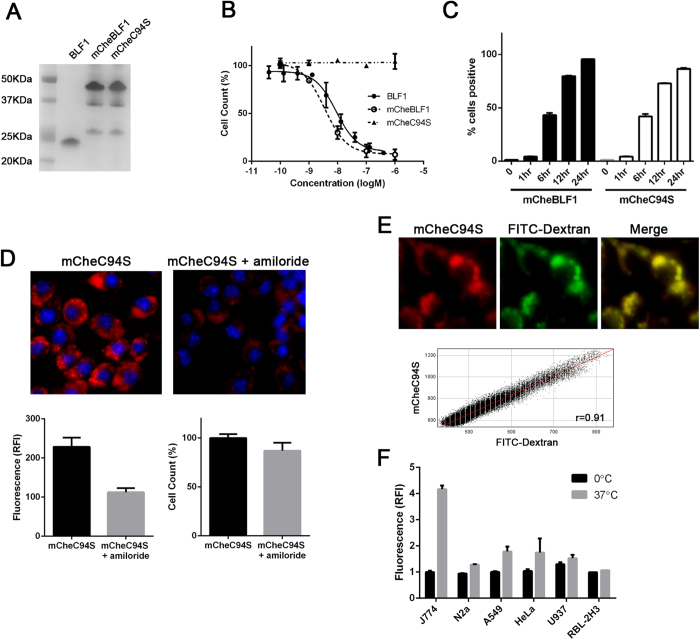
Cytostatic effect of BLF1 is due to its enzymatic activity and may be associated with macropinocytosis in the macrophage J774.2 cell line. (**A**) Coomassie-stained gel showing BLF1, mCherry-fused BLF1 and C94S inactive mutant. (**B**) Titration curves showing that fusing mCherry to BLF1 still permits cytosolic entry and activity whereas a single cysteine to serine mutation abolishes BLF1 cytostatic effects. (**C**) Bar chart showing uptake of the mCherry-BLF1 proteins at indicated times measured by flow-cytometry. (**D**) Fluorescence microscopy images showing decreased uptake of mCheC94S (1 μM) in the presence of 1 mM amiloride. The bar charts show a 50% reduction in uptake of mCheC94S in the presence of 1 mM amiloride (lower left panel) with little effect on viability (lower right panel). (**E**) Representative images showing strong colocalization of mCheC94S with FITC-dextran (70 kDa) with the cytofluorogram demonstrating high level of colocalization between mCheC94S and FITC-dextran (Pearson correlation coefficient, *r* = 0.91, SEM: ±0.006). (**F**) Flow cytometry of FITC-dextran uptake for 1 hour at 37 °C or on ice (0 °C) in different cell lines demonstrates the uniquely high level of dextran uptake by J774.2 cells. All experiments were in triplicates, mean ± SEM.

To examine the mode of uptake of proteins by J774.2 cells, we made mCherry-tagged BLF1 constructs including an enzymatically inactive C94S mutant^13^ (Fig. 4A). Titration experiments show that the mCherry-fused BLF1 enzyme exhibits the same level of growth inhibition as the non-tagged version (Fig. 4B) The increase in size of the protein from 24 kDa to 50 kDa did not affect BLF1 action, suggesting that protein uptake and entry into the cytosol are size independent, at least in this protein range. The catalytically inactive C94S mutant did not interfere with cell growth, confirming that growth inhibition is due to the BLF1 enzymatic action rather than general protein uptake. Analysis by flow cytometry demonstrated that mCherry-labelled proteins accumulated gradually inside cells and were present at detectable levels in almost all J774.2 cells by 24 hours, suggesting a non-specific fluid phase uptake that might be indicative of macropinocytosis (Fig. 4C). Macrophages are known to exhibit not only phagocytosis but also macropinocytosis, a form of non-selective uptake of fluid and solid cargo, which plays a major role in antigen presentation^14^. We therefore investigated the effects of amiloride, a known inhibitor of macropinocytosis, on the internalisation of the mCherry-labelled BLF1 C94S^15^. Pre-incubation of J774.2 cells with 1 mM amiloride for 30 min followed by a 4 hour incubation with mCherry-tagged protein showed a strong decrease in internalisation compared to the untreated control (Fig. 4D, 50%, p < 0.05). Next, we carried out co-localization studies with FITC-tagged dextran (70 kDa), a fluid phase marker commonly used in macropinocytosis studies^16^. Fluorescence microscopy showed almost complete co-localisation of mCherry-tagged C94S mutant with FITC-dextran (Fig. 4E), indicating that internalisation of C94S takes place via macropinocytosis. Quantification of FITC-dextran internalisation by flow-cytometry revealed that J774.2 cells exhibit a five times higher uptake activity compared to other tested cell lines at 37 °C (Fig. 4F). The enhanced macropinocytic activity of the J774.2 macrophage cell line may explain their acute sensitivity to externally applied enzymes.

**Figure 5:**
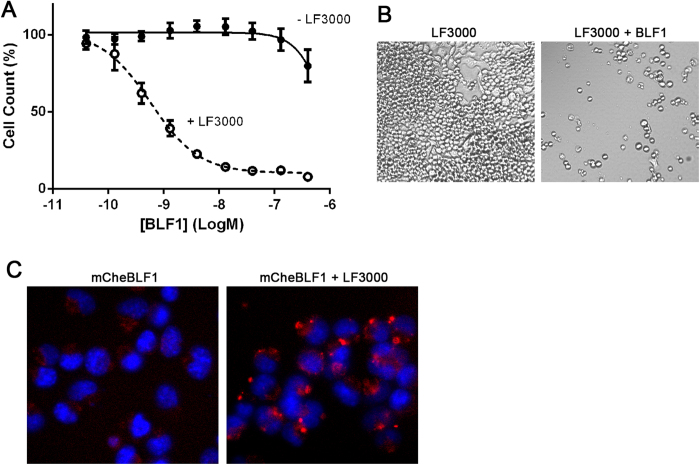
BLF1 enzyme arrests growth of neuroblastoma N2a cells. (**A**) Titration of BLF1 in the absence and presence of LF3000 indicates EC_50_ ≈ 1 nM for the intracellular activity of this enzyme at 72 hrs. (**B**) Representative images of the N2a tumour cells treated with LF3000 in the absence (left panel) or presence of 30 nM BLF1 (right panel) demonstrate an arrested state with BLF1 after 68 hrs (full time-lapse microscopy is in Supplementary information). (**C**) Representative images showing enhanced uptake of mCherry-BLF1 by N2A cells in the presence of LF3000. Punctate vesicular staining is evident in all cells treated with LF3000. All experiments were in triplicates, mean ± SEM.

The cytostatic nature of BLF1 observed in J774.2 cells could be important in biomedical applications. To confirm that the blockade of translation initiation by BLF1 can arrest cell growth without causing cell death, we analysed its effects on the neuroblastoma N2a cell line. As demonstrated in Fig. 1, these cells require LF3000 for cytosolic enzyme delivery. Incubation of the N2a cells with BLF1 in the presence of LF3000 for 72 hrs led to a dramatic inhibition of neuroblastoma growth at 1 nM concentration (Fig. 5A). Time-lapse microscopy confirmed that BLF1 significantly retards neuroblastoma cell growth with no apparent loss of cellular integrity (Fig. 5B and Supplementary movies). Following 24 hr incubation of mCherry-BLF1 with LF3000, the fluorescent protein was highly enriched in segregated dense vesicle-like structures near nuclei in the vast majority of the N2a cells (Fig. 5C). The observed cytostatic effects of BLF1 at low nanomolar concentrations indicate that LF3000 not only concentrates proteins in the vesicular structures but also induces leakiness of the endocytic membranes to achieve its transfection/transduction effects.

## Discussion

In this study we demonstrated that the recently-introduced lipofection reagent LF3000 allows efficient delivery of enzymes into a range of cancer cells. We also demonstrated that the macrophage J774.2 cell line is uniquely penetrable to intracellularly-active enzymes. The important advantage of these two methods for protein delivery is that they offer a more uniform intracellular introduction of enzymes compared to microinjections or DNA-based protein expression. The mechanisms underlying the advantageous characteristics of LF3000 over other lipofection and proteofection reagents are currently unclear due to proprietary reasons. Its universal availability, nevertheless, will allow cross-referencing with other cells and biological macromolecules. The unique penetrability of the J774.2 cells raises important questions for future investigations. We have shown that J774.2 cells exhibit enhanced macropinocytosis, and indeed a recent study shows that induction of macropinocytosis can help to drive intracellular delivery of native proteins^17^. However, it would be interesting to determine how exactly the enhanced uptake via macropinocytosis may account for the unusual sensitivity of J774.2 cells to the cytosolic action of externally applied enzymes. We speculate that protein translocation into the cytosol of J774.2 macrophages occurs during cross-presentation, which can involve export of polypeptides from endocytic-like compartments into the cytosol for degradation. Despite intense research over decades, mechanistic details of how uptaken proteins are transported into the cytoplasm are still under investigation^18^. It will be interesting to determine whether primary macrophages and other macrophage cell lines have a similar sensitivity to exogenous proteins. Regardless of the exact nature of cytosolic entry, the two presented methods provide a useful platform to start addressing the intracellular action of various enzymes without the need for complicated protein delivery techniques such as microinjection or specialised and costly peptide-based approaches^5^,^6^.

Our investigation revealed that botulinum-derived enzymes can affect the biology of immune cells by cleaving a single protein, VAMP3. Further, we showed that among translation-inhibiting enzymes, saporin exhibits the highest efficacy which is, however, associated with cytotoxicity. One issue with saporin and other ribosome-inactivating proteins is their high potency against all cells, making precise targeting to tumour cells of paramount importance. Since antigens expressed solely on tumour cells are rare, hepatotoxicity and capillary leak syndrome are common with these forms of treatment^8^. By contrast, the recently discovered BLF1 enzyme, which targets the eIF4A initiation factor, was able to block cell growth with little cytotoxicity. Blocking initiation of translation has recently gained considerable attention^19^ and our data on BLF1 action may be relevant to the design of new anti-cancer therapies. In addition to cancer treatment, blocking initiation of translation in neurons has recently been suggested as a possible therapeutic route for pain relief and other neurological disorders^20^. Our new data on neuroblastoma cells makes BLF1 an interesting candidate for inhibiting protein translation in pain-related neurons. These findings illustrate the effectiveness of the two complementary methods for assaying intracellular actions of exogenous enzymes and open new avenues for high-throughput screening and quality control in biotechnology.

## Methods

### Cell Culture

Cell lines were maintained in a 37 °C incubator at 5% CO_2_. The N2a (ATCC), J774.2 (ECACC) and A549 (ECACC) cell lines were cultured in Dulbecco’s Modified Eagle Medium (Gibco) supplemented with 10% fetal bovine serum (FBS) (Gibco). SH-SY5Y cells (Sigma-Aldrich) were cultured in a 1:1 ratio of Eagle’s Minimum Essential Medium (Gibco) and F12 Medium (Gibco) supplemented with 1% non-essential amino acids (Gibco) and 15% FBS. HeLa cells (ECACC) were cultured in EMEM with 10% FBS, U937 cells (gift from Dr Jim Gallagher) were cultured in RPMI (Gibco) with 25 mM HEPES (Gibco) and 10% FBS, and RBL-2H3 cells (gift from Dr Birgit Helm) were cultured in DMEM (Gibco) with 1% non-essential amino acids and 10% FBS. Cells were plated at a density of 3 × 10^4^ cells per well in uncoated 48-well plates (Corning) or at 5 × 10^3^ cells per well in uncoated 96-well plates (Corning). For microscopy, 96-well plates with a μClear base were used (Greiner Bio-one) for better resolution.

### Enzymes and Protein Transduction

Botulinum protease type D-encoding DNA fragment (aa 1-446) was inserted into pQE3 (Qiagen) using EcoRI and SalI sites. Complementary oligonucleotides encoding the histidine affinity tag PPTPGHHHHHH followed by a stop codon were ligated into the EcoRV site to yield pBN31. The botulinum protease was produced in *E.coli* M15pREP4 strain (Qiagen) following 3 h of induction at 21 °C and purified on Ni^2+^-nitrilotriacetic acid agarose. Fractions containing the botulinum protease were pooled and dialyzed against 150 mM potassium glutamate, 10 mM HEPES-KOH, pH 7.2, frozen in liquid nitrogen, and kept at −70 °C. Saporin from *Saponaria officinalis* seeds was obtained from Sigma-Aldrich. BLF1 was purified as previously described^13^. BLF1 was cloned as a PCR fragment into pET14b (Novagen) using the NdeI and BamHI sites, allowing expression of N-term 6xHis tagged BLF1. 6xHis-mCherry-tagged BLF1 was built following two successive steps: (i) BLF1 was first cloned as a XbaI PCR fragment into pGEX KG-mCherry construct^21^, (ii) mCherry-BLF1 was then cloned as a NdeI/BamHI PCR fragment into pET14b (Novagen). Both 6xHis and 6His-mCherry tagged BLF1 were expressed into *E. coli* BL21-RP cells following overnight induction at 20 °C with 0.4 mM IPTG. Recombinant proteins were purified using Ion Metal Affinity Chromatography using TALON/Co2+ beads (Clontech) and then on Superdex 200 gel filtration column (GE Healthcare). Ricin chain A-encoding DNA fragment (aa 36-302 from *Ricinus communis*) was inserted into PGEX-KG using *EcoR*I and *Nco*I sites and was purified from *E.coli* DE3 *pLys* strain after 16 hr expression at 20 °C, using glutathione-affinity chromatography followed by thrombin elution and gel-filtration as described^21^. The pET-15b LFN-DTA plasmid (Addgene 11075), carrying DNA encoding the diphtheria toxin-derived enzyme (aa 33-222 from *Corynephage beta*), was modified to remove the LFN part by digestion with *Nco*I restriction enzyme. The diphtheria enzyme was purified as described previously^22^. Protein concentrations were estimated by SDS-PAGE (12% NuPAGE, Life Technologies) and Coomassie Blue staining with bovine serum albumin in a serial dilution used as a standard. Enzyme transduction was performed 6 h after plating using Lipofectamine LTX (Invitrogen), Lipofectamine 3000 (Invitrogen) and Proteofectene (Biontex). Proteins were pre-incubated for 20 minutes at 20 °C in 100 μl Optimem (Gibco) with or without 2.5 μl of the transfection reagents, with 10 μl (for 96-well) or 30 μl (for 48-well) being added to the wells. Cells were incubated at 37 °C and 5% CO_2_ with the indicated proteins and transfection reagents for 68–72 h before being assayed.

### Assays for cell viability, apoptosis, VAMP3 cleavage, flow cytometry and microscopy

Cell numbers were analysed using the Cell Counting Kit-8 (CCK-8; Sigma-Aldrich) according to manufacturer’s instructions. Briefly, the CCK-8 reagent was added in a 1/10 ratio and incubated at 37 °C for 1 h. Absorbance was then measured at 450 nm using a FLUOstar OPTIMA plate reader (BMG Labtech). Readings observed in the absence of any delivery reagent were taken as 100% after normalisation to a blank control (cell-free medium with CCK-8 reagent). To evaluate nuclear morphology, live cells were stained with Hoechst 33342 (1 μg/ml, Invitrogen) for 20 minutes at 37 °C and observed using a digital fluorescence microscope (DMIRB, Leica Microsystems). Live cellular uptake of mCherry proteins was visualised using the inverted fluorescence microscope. Caspase 3/7 activity was measured using the CellEvent Caspase 3/7 Green detection kit (Invitrogen) according to manufacturer’s instructions. Briefly, the CellEvent reagent was added to cells at a final concentration of 5 μM. Cells were then incubated for 30 minutes at 37 °C before imaging under a fluorescence microscope. Green fluorescent nuclei were indicative of caspase 3/7 activation and apoptosis. Images were taken of three different fields of vision for each well at 40 × magnification. Cells were then counted using the ImageJ software and the number of apoptotic cells was given as a percentage of total cell count. For analysis by flow cytometry, cells were seeded at 5 × 10^5^ in 6-well plates and incubated in medium containing 100 μg/ml mCherry-labelled BLF-1 proteins for up to 24 hours. Dextran uptake was quantified by incubation with 100 μg/ml FITC-dextran for 1 hour at 37 °C or on ice. After washing, cells were harvested and analysed using a LSRII flow cytometer (Becton Dickinson) and FlowJo software. In amiloride experiments, J774.2 cells were allowed to attach for 24 hours in 96-well μClear plates. Cells were pre-treated for 30 minutes with 1 mM amiloride (Sigma-Aldrich) before 4 hour incubation with 1 μM mCheC94S. For quantification, five images were taken at 60 × magnification followed by analysis of cytoplasm fluorescence intensity using ImageJ. For dextran colocalisation studies, cells were incubated with 1 μM mCherry-C94S and 1 μM FITC-Dextran for 4 hours before fluorescence microscopy and analysis using ImageJ with the JACoP Plugin^23^. Western Immunoblotting using anti-VAMP3 antibody (gift from Andrew Peden) was carried out as described previously^7^. Time-lapse live imaging was carried out using the JuLI FL live cell movie analyser (NanoEnTek). After application of proteins, cells were imaged every 20 minutes for 68 h at 37 °C.

**Supplementary movies.** 68 hrs time lapse videos of the N2a tumour cells treated with LF3000 in the absence (Movie 1) or presence of 30 nM BLF1 (Movie 2) were taken to demonstrate growth arrest only with BLF1.

## Additional Information

**How to cite this article**: Rust, A. *et al.* Two complementary approaches for intracellular delivery of exogenous enzymes. *Sci. Rep.* **5**, 12444; doi: 10.1038/srep12444 (2015).

## Supplementary Material

Supplementary Information

Supplementary Information
